# Spanish Science Teacher Educators’ Preparation, Experiences, and Views About Nature of Science in Science Education

**DOI:** 10.1007/s11191-021-00263-6

**Published:** 2021-09-16

**Authors:** Antonio García-Carmona

**Affiliations:** Departamento de Didáctica de Las Ciencias Experimentales Y Sociales, Universidad de Sevilla, Seville, Spain

## Abstract

The obstacles and difficulties that science teachers face when teaching the nature of science (NOS) are well-known. Nonetheless, little is known of what science teacher educators (STEs) know, do, and think about this issue. Thus, a study with 142 Spanish STEs was conducted. This was aimed at analysing (*i*) what preparation the STEs have to teach NOS, (*ii*) what educational experience they have about NOS, (*iii*) what importance they give to NOS in their training plans, (*iv*) when NOS should be taught, (*v*) how to integrate NOS in science education, (*vi*) how to teach NOS, (*vii*) what to teach about NOS, and (*viii*) the influences interconnecting the STEs’ preparation, experience, and opinions regarding NOS teaching. Among the results, it was found that most STEs state that they have ample knowledge of NOS, are well prepared pedagogically to teach it, and have extensive experience in teaching it. However, there was some mismatch between their stated preparation and their experience teaching NOS. In addition, they think that NOS should be taught from early ages and be treated as cross-cutting content in science education. They also consider that, when teaching NOS, an implicit approach is just as effective as an explicit-reflective one. As for their selection of NOS content to include, they are fundamentally divided into two groups — those who select only epistemic aspects of NOS and those who select a balanced proportion of both epistemic and non-epistemic aspects. The limitations of the study are reported along with the issues that require further research.

## Introduction

The meta-scientific understanding of the characteristic features of science, such as its purposes, practices, methods, limits, values, organization systems, and interactions with society, is accepted as being a key dimension of scientific literacy in citizenship (National Science Teaching [NSTA], 2020; Organisation for Economic Co-operation and Development (OECD), 2019). As meta-knowledge, it is polyhedral and dynamic (i.e. in continuous revision), constructed mainly with contributions from the philosophy, history, and sociology of science. The most common term used for it is *nature of science* (NOS) (Acevedo-Díaz & García-Carmona, 2016; McComas & Clough, 2020).

In addition to favouring the understanding of scientific knowledge (Driver et al., 1996; Michel & Neumann, 2016; Nelson et al., 2019), informed knowledge about NOS is especially useful for putting forward opinions, evaluating and then making responsible decisions in public matters related to science (Allchin, 2014; Miller, 2004; Sadler et al., 2004). Indeed, according to Shamos (1995), when people opine on public science issues, they usually base their arguments on their conceptions about NOS. Certainly, the robustness or scientific soundness of people’s arguments in relation to such issues depends mostly on how comprehensively informed their NOS conceptions are. Therefore, as noted by Driver et al. (1996), the integration of NOS content into the school science curriculum has a utilitarian, democratic, cultural, axiological, and educational justification. For this reason, the basic notions about NOS are suggested as being essential content from the early levels of science education onwards (Akerson et al., 2011; Cañal et al., 2016; NGSS Lead States, 2013).

Nonetheless, there is still a long way to go before the comprehension of basic aspects of NOS is consolidated as an important objective in the science education promoted in the classroom. Apart from the epistemological and ontological complexity inherent in the NOS construct (Acevedo-Díaz & García-Carmona, 2016), there are multiple reasons behind this educational situation. Two of them stand out. The first is the huge gap between, on the one hand, what is proposed (or omitted) in the standard documents of science education, textbooks, etc., regarding the teaching of NOS and, on the other, the recommendations deriving from educational research in this respect (Ferreira & Morais, 2013; Höttecke & Silva, 2011; McDonald & Abd-El-Khalick, 2017; Olson, 2018). In Spain, for example, the official science curriculum for basic compulsory education (6–16 years) hardly mentions any aspects related to NOS in the proposals of content and evaluable learning standards (Acevedo-Díaz et al., 2017). Likewise, very little attention has been paid to NOS in Spanish scientific publications about science education during the last decade (García-Carmona, 2021c). In general, the teaching of NOS in Spain is an issue that still needs to be promoted and improved within the country’s science curricula and science teacher training programs (Acevedo-Díaz et al., 2017; Acevedo-Díaz & García-Carmona, 2016; Perales et al., 2014; Vázquez-Alonso et al., 2013).

The other notable reason corresponds to the various obstacles and difficulties science teachers face when teaching NOS (Acevedo-Díaz, 2008; Aslan & Taşar, 2013; Capps & Crawford, 2013; Clough et al., 2020; Garcia-Carmona et al., 2011; Piliouras et al., 2018). Undoubtedly, the lack of an informed comprehension of NOS favours neither science teachers deciding to include NOS content in their science teaching programs nor their knowing how to teach it appropriately (Vázquez-Alonso et al., 2013; Sarieddine & BouJaoude, 2014). Even if a science teacher does possess an informed understanding of NOS, this is no guarantee that they will choose to introduce it into their classes or that they will know how to do so properly (Akerson & Abd-El-Khalick, 2003). It may be that they do not have the confidence, the support, and/or the sufficient educational preparation to teach NOS (Deniz & Adibelli, 2015; Sarieddine & BouJaoude, 2014; Supprakob et al., 2016).

It may also happen that science teachers simply decide not to deal with NOS content in their classes because their educational convictions lead them to prioritize other science content over NOS (Waters-Adams, 2006). Indeed, this last may even be reasonable if, for example, NOS is not explicitly the object of teaching and evaluation in the standard science education documents that the teacher handles (García-Carmona, 2021c). This is therefore a complex situation that still requires major attention from science education research as well as education policy makers.

As indicated above, there is currently a wealth of information available about the most common obstacles and difficulties faced by science teachers when teaching NOS. Nonetheless, very little is known about what science teacher educators (hereafter referred to as STEs) know, do, and think about NOS in science education. Similar to the case with science teachers, STEs’ preparation and interests with regard to teaching NOS will condition the level and mode of attention they give to it in their science teacher training plans (Irez, 2006; Wan et al., 2011). In addition, because STEs are a fundamental piece in the training and professional development of science teachers training in relation to NOS teaching, having information about what STEs think and do at this respect within their training plans can also help to understand in part the problems noted before. Consequently, it was decided to address this question through an exploratory and interpretive study with a representative sample of STEs in the educational context of Spain.

## Theoretical Framework

### Selection of NOS Content for Science Education

The breadth and multifaceted nature of NOS as meta-knowledge mean that determining which aspects of it should be taught is a complex question in permanent debate among the international science education community (Acevedo-Díaz & García-Carmona, 2016; Allchin, 2011; Hodson & Wong, 2014; Matthews, 2012; Wallace, 2017). Despite this, one of the proposals of NOS content to teach has dominated the international scene over the last two decades. This is the proposal put forward by Lederman (2007). It focuses essentially on the understanding of epistemic (i.e. rational or cognitive) features of science, such as differences between scientific law and theory or between observation and inference, the theoretical underpinnings accompanying all scientific observations and interpretations, the tentative nature of scientific knowledge, and the methodological plurality of science. The contextual, social, and psychological aspects involved in the development of science (i.e. non-epistemic aspects of science) hardly receive any attention in that vision of NOS, except for a very generic allusion to the fact that the construction of scientific knowledge is influenced by the cultural and social context (and vice versa).

Nonetheless, when the history, philosophy, and sociology of science have been reviewed in depth, it has been found that multiple non-epistemic factors also decisively influence its development (Elliott & McKaughan, 2014; García-Carmona, 2021a; García-Carmona & Acevedo-Díaz, 2018; Knorr-Cetina, 1981; Parker & Winsberg, 2018; Pournari, 2008). Consequently, it is reasonable that understanding these aspects should receive similar attention to that of epistemic aspects in programming teaching NOS. In recent years, there have been some interesting approaches to teaching NOS with this wider perspective (e.g. Allchin, 2011; Dagher & Erduran, 2016; García-Carmona & Acevedo-Díaz, 2018; Irzik & Nola, 2014). For example, Irzik and Nola (2014) propose that the understanding of NOS should include both cognitive-epistemic factors (research processes and objectives; values such as prediction, explanation, consistency, simplicity, and utility; and methodological procedures and rules) and socio-institutional factors (scientists’ professional activities; scientific ethos; certification and dissemination of scientific knowledge; and science’s social values). Similarly, Dagher and Erduran (2016) stress the importance of organizations and social interactions, public power structures, and science financing. To various aspects of an epistemic nature, Allchin (2011) adds the attention to social interactions among scientists and conflicts of interest and ethics in science as a, step towards proposing a fuller assessment of understanding NOS. From a perspective similar to that of the aforecited authors, García-Carmona and Acevedo-Díaz (2018) suggest a holistic form of teaching NOS which addresses both epistemic and non-epistemic aspects of science in a balanced way:

- *Epistemic aspects of NOS:* (*i*) nature of science processes (influence of scientists’ beliefs and abilities in their research, role of models and modelling in science, observation vs inference, role of questions and hypotheses in science, role of error in science, relationships between research designs and empirical results, methodological diversity in scientific research, etc.) and (*ii*) nature of scientific knowledge (differences between scientific laws and theories, provisional nature of scientific knowledge, etc.)
- *Non-epistemic aspects of NOS:* (*i*) factors internal to the scientific community (role of scientific communication, scientists’ personality and motivation, gender in science, scientific collaboration and competitiveness, professional and personal relationships among scientists, etc.) and (*ii*) factors external to the scientific community (political, economic, and cultural influences on science and vice versa; science and religion; etc.)

Therefore, alternatives such as this extend somewhat closed NOS content proposals such as that of Lederman (2007) and constitute a broader and more diverse framework with a view to selecting aspects of NOS for the science classroom that are in accord with each situation, context, or educational need. This may contribute to favouring the introduction of NOS into science education (Acevedo-Díaz et al., 2017).

### Pedagogical Approaches and Strategies for Teaching NOS

With respect to how NOS should be taught, empirical research (Clough, 2018; Deniz & Adibelli, 2015; Khishfe & Abd-El-Khalick, 2002; Lederman, 2007) has shown that the best way to learn NOS is through an explicit and reflective didactic approach. This means that NOS should be regarded as (*i*) specific curricular content with its own learning objectives and that its implementation in class needs (*ii*) a design of activities that foster pupils’ reflection about and discussion of NOS issues and (*iii*) a specific plan for evaluating the pupils’ achievements and learning difficulties (García-Carmona, 2021b; Schwartz et al., 2004).

Nonetheless, according to the exhaustive review of the literature by Acevedo-Díaz (2009) on this question, there are still some who argue that understanding aspects of NOS may also be attained through an implicit or indirect approach.^1^ An implicit approach assumes that the construction of learning about NOS is a natural consequence of the simple fact of participating in school scientific inquiry activities (Schwartz et al., 2004). However, such an approach is hard to sustain after educational research had already and repeatedly found it to be ineffective as against the explicit-reflective approach (Acevedo-Díaz, 2009; Lederman, 2007). A possible cause of this inefficacy is that the implicit approach induces a naive identification of the understanding of NOS with carrying out science processes in inquiry activities (Acevedo-Díaz, 2008; García-Carmona et al., 2011; Lederman, 2019). Indeed, pupils can acquire basic skills to make measurements in the context of a scientific inquiry, but if they do not reflect and then assimilate that this practice has an intrinsic limitation imposed by the senses and measuring instruments used and is also influenced by their own skills, knowledge, and expectations, they will not have learnt about the nature of scientific measurement. Hence, it is important to pose specific questions that explicitly invite the pupil to reflect and think about it.

Moreover, a review of the literature about how NOS content is usually introduced into school science curricula (Acevedo-Díaz & García-Carmona, 2016) found the following ways to be used: (*i*) NOS integrated with other school science content; (*ii*) NOS as independent content (or not integrated with the other curricular content); and (*iii*) through a combination of the two strategies. Some studies indicate that pupils’ understanding of NOS is independent of whether or not it is integrated with the rest of the science curriculum (Khishfe & Lederman, 2007). Integration, however, may have the advantage of hardly altering at all the planned program for a school science course, and this would encourage science teachers to introduce NOS content into their classes (Bell et al., 2012). Likewise, reflection about NOS content in authentic scientific development contexts, such as historical or contemporary scientific controversies on a given (social) scientific topic, can favour a more realistic vision of scientific activity (Acevedo-Díaz et al., 2017; Allchin, 2011; Clough, 2006; García-Carmona & Acevedo-Díaz, 2016).

On the other hand, it is worth noting that an informed understanding of NOS can be the basis or inspiration for establishing pedagogical principles that promote a form of science education which is more consistent with the practice of science (García-Carmona & Acevedo-Díaz, 2018). For example, Domin (2009) proposes inquiry-based science learning taking the Kuhnian vision of NOS as referent. Stephens and Clement (2012) note the form of research that some illustrious figures in the history of science applied and that such thought experiments might be adaptable to the classroom context as an interesting strategy for learning science. Adúriz-Bravo (2013) suggests taking the “semantic” view of scientific models as a framework in which to foster the practice of modelling in science education. In analogy with scientific evaluation, García-Carmona (2020) proposes that pupils submit their results of an inquiry to criticism through a combination of assessment by “anonymous” peers and “known” peers. In this way, in addition to enriching their conclusions with other points of view and comments, they can reflect on which of the two processes would be best in order to avoid conflicts of interest in an inquiry.

Finally, it should be noted that having informed knowledge about NOS is no guarantee that science teachers will choose to introduce it into their classes (Akerson & Abd-El-Khalick, 2003; Sarieddine & BouJaoude, 2014). But it is commendable to think that a robust knowledge of NOS at least provides some interesting references to promote authentic environments for science learning (Abd-El-Khalick, 2013). Some studies (Calagua et al., 2016; Dogan et al., 2013) have made it clear that, with an appropriate training program, science teachers will be able to transfer their understanding of NOS to their general teaching practice, i.e. to improve their pedagogical content knowledge (PCK) to teach school science. In this sense, the basic ideas of NOS should equally be a key factor in shaping a school science curriculum that is more in line with how science works (Dagher & Erduran, 2016; Hipkins, 2012; McComas, 2017).

Mentioned proposals suggest that science teachers’ training in NOS teaching is an ambitious and complex challenge, in which STEs constitute a key piece. But what is really known about what STEs know, do, and think about this issue? At least in the Spanish educational context, very little is known about it. That is why it was relevant to conduct this study.

## Research Questions

In light of all the above, the research questions that guided this study were the following:

1. What preparation do the STEs claim they possess regarding the teaching of NOS?
2. What experience do the STEs claim they possess in teaching NOS and in teaching NOS pedagogy?
3. What opinions do the STEs have about teaching and learning NOS?
4. What correlations exist between the STEs’ preparation, experience, and opinions about teaching and learning NOS?

## Methods

### Participants

The STEs of Spanish public universities were the object population to be studied in this research. The process followed for the selection of participants was the following. First, the web pages of the 50 Spanish public universities were consulted to locate those offering degrees related to teacher education. Next, it was checked whether specific science education departments were involved in these degree courses or, failing that, general education departments that included “science education” as an area of knowledge. Once located the departments or subsections dedicated to science education, the email addresses of the STEs that comprised them were acquired. The selection therefore was of those Spanish STEs whose information was clearly available on the public universities’ websites. The result of this process was a sample of 386 STEs belonging to 32 Spanish public universities.

The STEs were invited by email to participate in the study. Of the total of STEs selected (386 in total), only 142 responded to the survey, constituting 36.8% of the initial sample. The profile of the final sample of participants is detailed in Table 1.

**Table 1:** Characteristics of the science teacher educators (STEs) forming the study sample

*N* = 142			
Gender	Women: 69 (48.6%) Men: 73 (51.4%)		
Age	Range: 25–69 years Median: 47 years Mode: 53 years		
University degree (bachelor’s)	Experimental sciences: 88.0% Engineering: 7.0% Education sciences: 5.0%		
Doctorate	Science education: 88.0% Other/no doctorate: 12.0%		
Science teaching experience	None: 7.7% 1 to 5 years: 19.0% 6 to 10 years: 11.3% 11 to 15 years: 15.5% 16 to 20 years: 12.7% > 20 years: 33.8%	Educational stages *	Pre-primary (3–6 years): 1.4% Primary (6–12 years): 4.9% Lower secondary education (12–16 years): 29.6% Upper secondary education (16–18 years): 33.8% University: 83.1%
Experience as a science teacher educator	None: 1.4% ** 1 to 5 years: 28.9% 6 to 10 years: 27.5% 11 to 15 years: 17.6% 16 to 20 years: 5.6% > 20 years: 19.0%	Science teacher collective ***	Pre-primary (3–6 years): 39.4% Primary (6–12 years): 66.2% Secondary (12–18 years): 54.9% University: 44.4%

*Some STEs have taught or are teaching science at more than one educational stage

**Doctoral students who are training to be science teacher educators

***Some STEs have trained or are training science teachers of different educational stages

### Data Collection Instruments

#### Survey

The intention with the survey was to obtain basic information with which to answer the research questions that had been posed. In its design, standard validity and reliability criteria were taken into account for this type of research instrument, as will be explained below.

Regarding the content validity of the survey, its design was made according to the theoretical framework exposed above. In this design process, they were also key factors the long teaching and research career of the present study’s author with respect to the teaching of NOS, and the opportunity of having the opinion of a colleague, who is also expert in NOS teaching and who revised the survey to make some recommendations on the content, writing, and organization of the items.

With respect to the construct validity of the survey, the first thing was to try to ensure the greatest participation possible on the part of the STEs. In this sense, particular effort was made to avoid the survey requiring a long time to complete. The survey comprised 10 items that combined ordinal (items 1–6) and nominal or categorical (items 7–10) variables. In accordance with the purposes of the research, in the survey design, the items were organized in 8 dimensions, such as is shown in Table 2.

**Table 2:** Structure and characteristics of the survey

Subject of analysis	Dimensions	Items	Type of item
STEs’ preparation (RQ1)	I. Level of knowledge (CK) about NOS	1, 2	Ordinal (Likert: 1, … 10)
	II. Level of pedagogical content knowledge (PCK) to teach NOS	3	Ordinal (Likert: 1, … 10)
STEs’ experience (RQ2)	III. Teaching experience in NOS and in NOS pedagogy	4, 5	Ordinal (Likert: 1, … 10)
	IV. NOS in science teacher training programs	6	Ordinal (Likert: 1, … 10)
STE’s opinions (RQ3)	V. When to teach NOS	7	Nominal polytomous (more than one option to choose)
	VI. How to integrate NOS into the school science curriculum	8	Nominal polytomous (only one option to choose)
	VII. How to teach NOS	9	Nominal polytomous (only one option to choose)
	VIII. What aspects of NOS to teach	10	Nominal polytomous (more than one option to choose)

*RQ, research question

It is necessary to clarify that the results of item 10 were recoded for certain of the study’s analyses (including those of validity and reliability). In this, the participants were asked to choose from an ample list of NOS aspects of the ten that they would give priority to in science classes. As well as counting the frequency of the various aspects of NOS selected by the participants, the responses were coded into three categories according to whether the NOS aspects chosen by the participant were (1) mostly epistemic; (2) mostly non-epistemic; or (3) a balanced mix of epistemic and non-epistemic (5:5 or 6:4).

Once data were available, the construct validity of the survey was statistically analysed. Since, a priori, the items were organized into 8 dimensions, a confirmatory factor analysis procedure was followed to determine the survey’s degree of construct validity (Knekta et al., 2019). The IBM SPSS Statistics Base 26 program package was used for all statistical calculations. A principal component analysis was carried out, followed by varimax rotation with Kaiser normalization. The number of factors to extract was set at 8, with elimination of small coefficients (< 0.35). The results confirmed the eight dimensions of the survey, with factor loadings ranging from 0.86 to 0.99. The resulting factorial model showed good sampling adequacy on the Kaiser–Meyer–Olkin (KMO) test (0.75) and gave a value of 0.00 (< 0.05) for Bartlett’s sphericity (López-Roldán & Fachelli, 2015).

As the survey was only applied once, its reliability was determined through an internal consistency analysis of the set of items (Tavakol & Dennick, 2011). For this, Cronbach’s *α* was calculated. The value of *α* for the remaining 10-item survey (Table 2) was 0.76. This coefficient was also calculated for the two dimensions of the survey with more than one item (dimensions I and III; see Table 2). The values obtained were 0.83 and 0.81, respectively. All these values of *α* indicate that the survey presents a degree of internal consistency that is between acceptable and good (Ursachi et al., 2015).

#### Open-Ended Questionnaire

To complement a part of the information obtained with the survey, an open-ended questionnaire was also used. The intention with this was to obtain further or complementary information on the introduction and selection of NOS content for science education, as well as on the approaches or strategies best suited to teaching NOS, which are mainly related to items 8, 9, and 10 of the survey. Therefore, the process followed in the design of the questions was similar to that which had been followed with the items of survey concerned; only now the participants were given the opportunity to explain their opinions or positions. Since the completion of the open-ended questionnaire required much more time than the survey, it was decided to invite only a reduced portion of the sample to respond to it. This selection was made as follows. On the one hand, a random choice was made of 20 of the STEs of the total sample who had published some specific work about NOS over the preceding decade. This information was available from a prior study of the literature about NOS teaching in Spain (García-Carmona, 2021c). Another 20 STEs were selected, also at random, from the remaining part of the sample. These 40 STEs were invited to participate by email. Only 9 of those invited finally responded to the open-ended questionnaire: (*i*) 4 women and 5 men; (*ii*) ages 27–69 years, mean 53 years; (*iii*) school science teaching experience 0–33 years, mean 17 years; and (*iv*) experience as a science teacher educator 2–34 years, mean 11 years. It should also be added that 3 of these 9 STEs stated in their responses that they had little experience in teaching NOS.

### Data Analysis

In addition to the calculations indicated above to determine the validity and reliability of the instruments, the following procedures were applied in the analysis of the results:

i) *Survey.* The results of the survey were analysed both descriptively and inferentially. For the descriptive analysis, percentage frequencies were calculated. For the inferential analysis, the results were first subjected to a statistical normality test. Given the size of the sample of participants in the study (*N* > 50), the Kolmogorov–Smirnov test was used. This indicated that, for a significance level of 0.05, the results of the survey were not normally distributed, so that non-parametric statistics should be used. Thus, in order to determine possible correlations between the different dimensions of the survey (research question 4), calculations of Spearman’s *ρ* were applied (Sandoval, 2010).
ii) *Open-ended questionnaire.* The standard procedures for exploratory studies of content analysis (Mayring, 2000) were taken into account. Nonetheless, as the number of STEs that responded to the questionnaire was small (only 9 STEs), the intention was not to look for patterns of responses but rather to look for possible reasons that might be behind the trends observed in some of the dimensions of the survey.

## Results

### What Preparation Do the STEs Claim They Possess Regarding the Teaching of NOS?

**Fig. 1:**
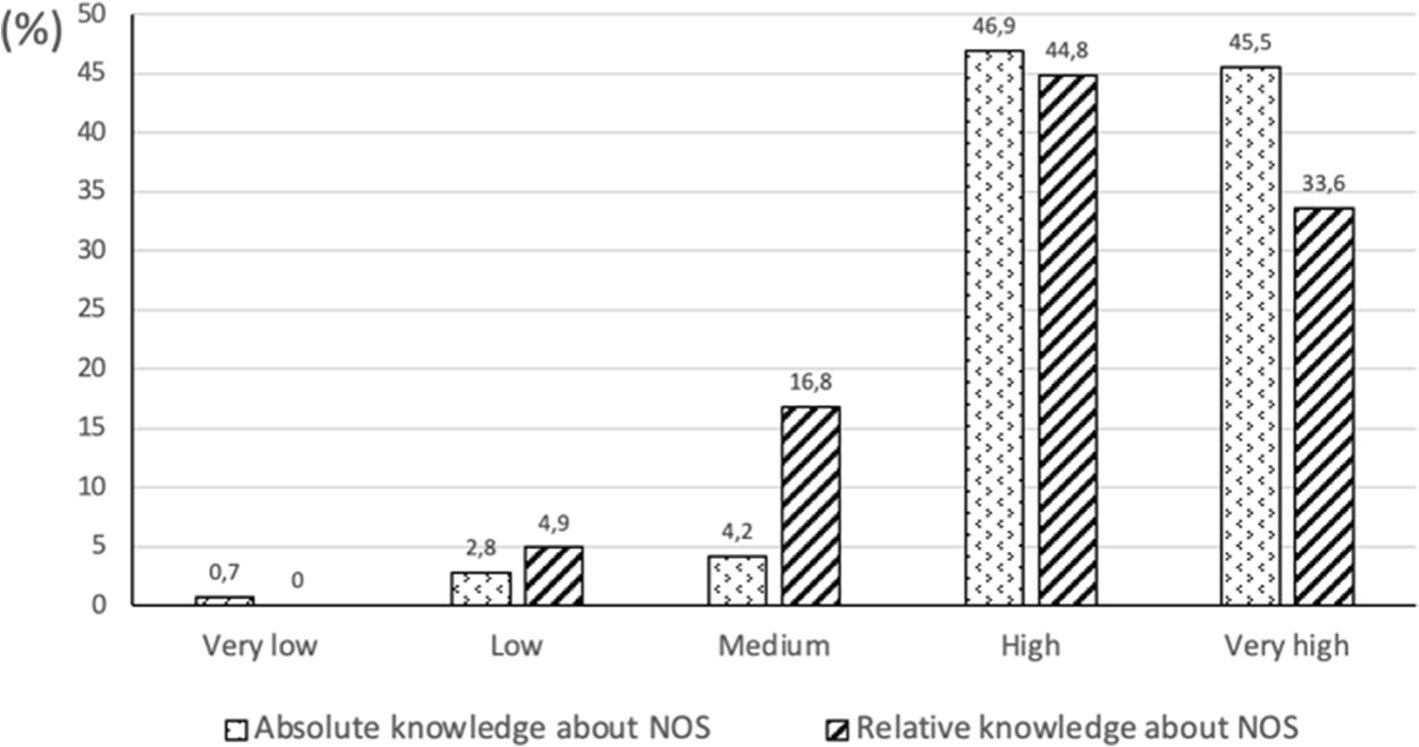
Levels of knowledge about NOS in absolute and relative (i.e. compared to other school science content) terms, self-estimated by the STEs

First, the STEs were asked to estimate their own level of content knowledge (CK) about NOS. The results are shown in Fig. 1. Most of the respondents indicated that their understanding of the subject was high (46.9%) or very high (45.5%). Only a very small portion indicated that they had a fairly limited knowledge of NOS (3.6%, summing the low and the very low levels). In order to complement this information, the STEs were asked to also assess their knowledge of NOS in comparison with their knowledge of other content from the school science curriculum (i.e. their relative understanding of NOS). It was found that the portion of STEs with the highest levels of understanding declined by 14 percentage points. In other words, the number of STEs with low and medium comprehension levels increased when they were asked to weigh their knowledge of NOS against that of other school science content. In total, just over a fifth of the STEs (21.7%) reported a medium to very low relative understanding of NOS.

**Fig. 2:**
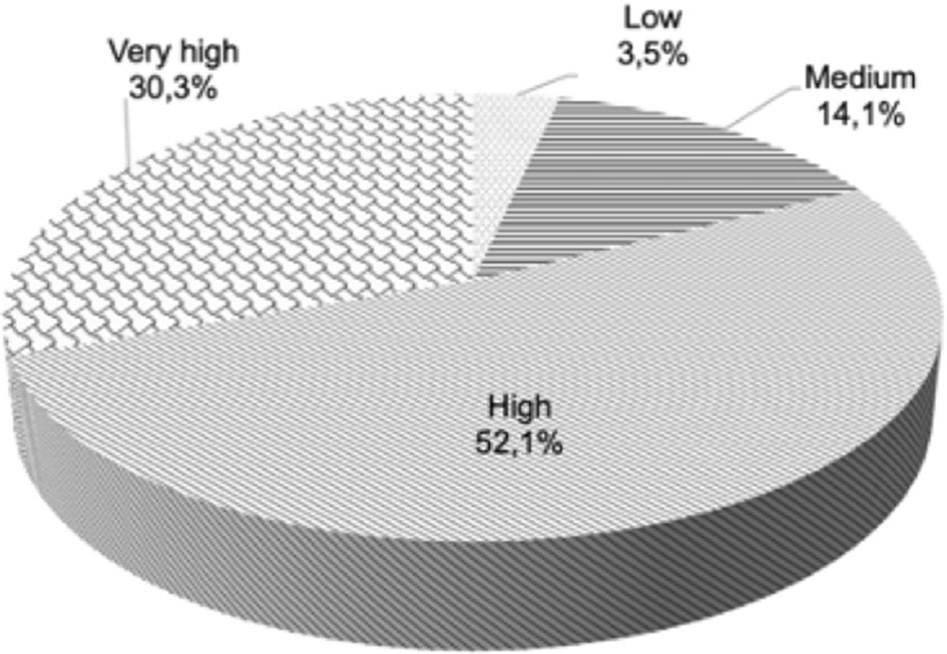
Levels of pedagogical content knowledge (PCK) for teaching NOS, self-estimated by the teacher educators

The STEs were also asked to give an overall assessment of their level of pedagogical preparation (i.e. their PCK) to teach NOS. The results are shown in Fig. 2. As can be seen, slightly more than half of the STEs estimated that their PCK for teaching NOS was high and a third that it was very high, while 14.1% stated that it was medium and a meagre 3.5% that it was low.

### What Experience Do the STEs Claim They Possess in Teaching NOS and in Teaching NOS Pedagogy?

#### Experience in Teaching NOS Content and in Training Science Teachers in NOS Pedagogy

**Fig. 3:**
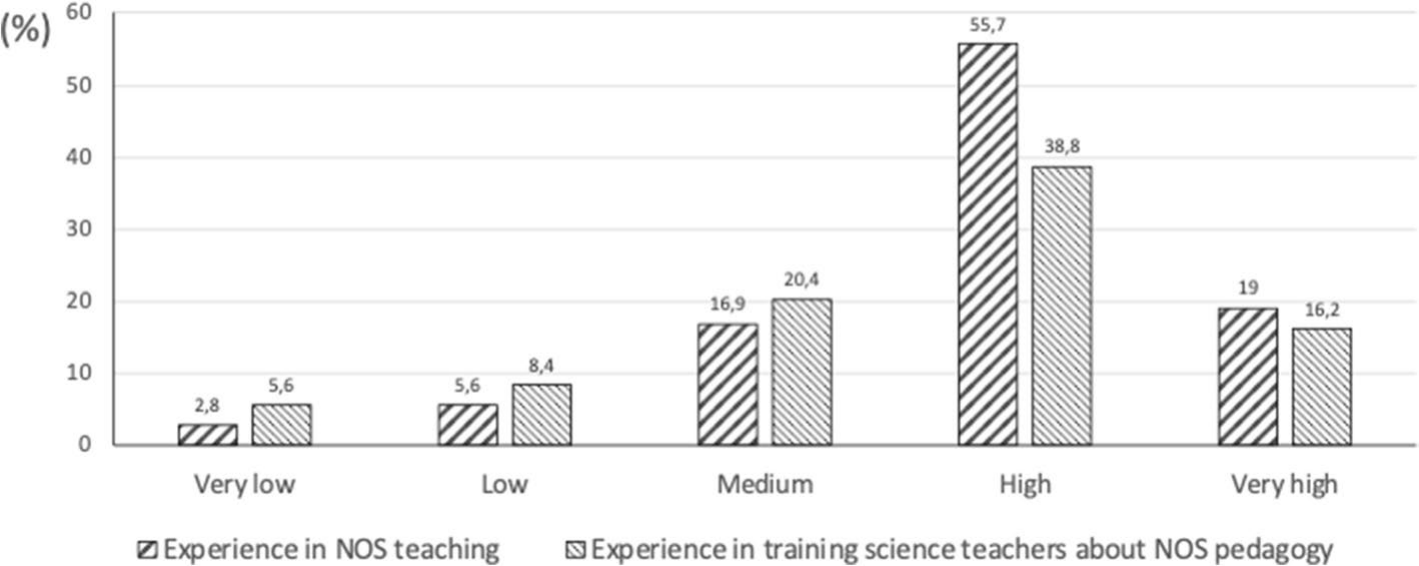
Experience in teaching NOS and about its pedagogy

Figure 3 shows the results of asking the STEs about their own experience in both NOS teaching (i.e. as science teachers who teach about NOS) and training science teachers in NOS pedagogy. What most stands out is that 55.7% of the STEs estimated their experience in teaching NOS content as being high, although when also asked about their experience as teacher educators who train science teachers in NOS pedagogy, only 38.8% declared their level as being high. In general, one observes that the STEs have more experience in teaching NOS content than in training science teachers about NOS pedagogy. Additionally, it is worth mentioning that 14% of the STEs stated that they had low or very low teaching experience in training science teachers in NOS pedagogy and that 8.4% had hardly any experience in teaching NOS content.

#### Attention to NOS in Science Teacher Training Plans

**Fig. 4:**
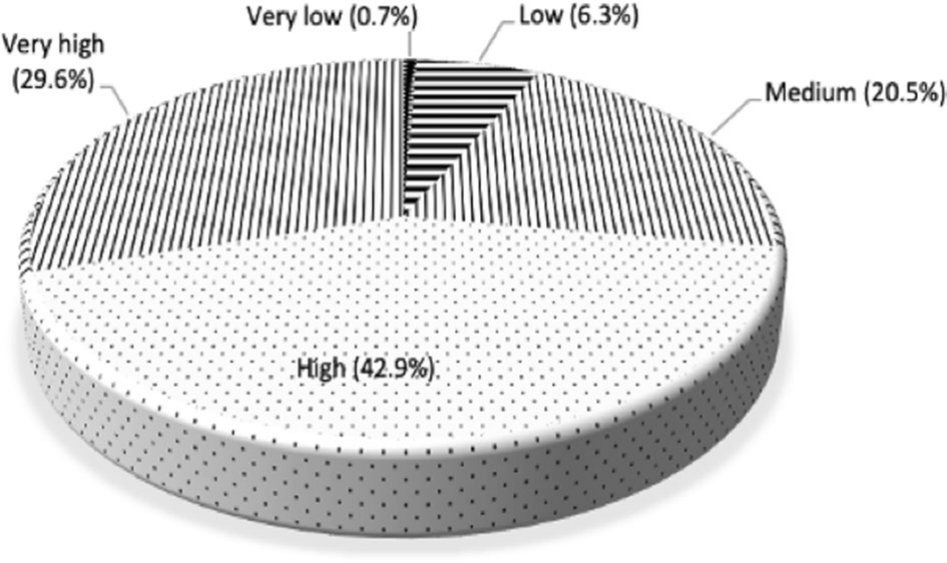
Attention or importance that STEs gave to NOS content in their science teacher training plans in comparison with other contents of the science curriculum

Another question explored was the degree of attention or importance that the STEs give to NOS in their teacher training plans. The results (Fig. 4) indicate that 42.9% of STEs give high importance to NOS compared to other science education content in science teacher training. This importance of NOS in the training plans was very high for 29.6% of the STEs. Nonetheless, it is also noteworthy that for just over a quarter of them (27.5%), the priority given to NOS content in their science teacher training plans ranged from medium to very low.

### What opinions Do the STEs Have About Teaching and Learning NOS?

#### Educational Stages at Which NOS Should Be Taught

When the STEs were asked about when NOS content should be introduced in science teaching, the main position taken was that it should be taught at all educational stages (43.7%). Nonetheless, around a quarter of the STEs thought that the stage of early childhood education should be excluded from this generalization of teaching NOS in science education. The rest were divided between those who considered that NOS should only be taught at specific educational stages, such as lower secondary education (9.9%) or only in post-compulsory educational stages (7%). The complete results of this question are listed in Table 3.

**Table 3:** STEs’ opinions on when to introduce notions of NOS in science education

NOS should be introduced…	in all educational stages, including pre-primary education	43.7%
	in all educational stages from primary education onwards	25.4%
	from lower secondary education onwards	9.9%
	only in post-compulsory educational stages	7.0%
	only up to and including lower secondary education	6.3%
	in all pre-university educational stages	3.5%
	or after options (e.g. only in the university stage or just in lower secondary education)	2.8%
Understanding NOS is not considered a primary educational goal at any stage		1.4%

#### Introduction of NOS Content into the School Science Curriculum

Another fundamental question in this study was to determine the STEs’ opinions about how to introduce NOS content into the school science curriculum. According to the results presented in Table 4, a vast majority of the STEs (82.4%) consider that NOS should be presented as cross-cutting content that is integrated with the rest of the science content in the curriculum. Only 12.7% of the STEs thought that NOS should constitute specific content, which would then give rise to its own didactic unit in science education programs. The rest of the STEs consider that NOS should be considered to be secondary or supplementary content compared with other more basic content of the science curriculum (3.5%) or simply believe that it should not be considered to be an essential content of the curriculum (1.4%).

**Table 4:** STEs’ opinions on how to introduce NOS into the school science curriculum

NOS as…	specific content of the science curriculum, constituting its own unit in school science programs	12.7%
	cross-cutting content, integrating it with the other contents of the school science curriculum	82.4%
	secondary or supplementary content to be dealt with in certain units of school science programs	3.5%
NOS is not considered to be essential content in the school science curriculum		1.4%

The above information was complemented through the open-ended questionnaire. When asking the STEs about how to introduce NOS content into the science curriculum, three different strategies were detected. In the first place, there are those STEs who incorporate NOS content into their teaching plans without integrating it with the other content of the programmed curriculum. The way some of them explain it is as follows:

[In each course] I try to schedule the first classes to explicitly deal with some NOS ideas, before relating it to some other school science topic. Normally, [I propose to the students] some activity that also serves to ‘break the ice’ (…). (STE-5; brackets added)

I explicitly include NOS contents in a lesson about the history and philosophy of science within a subject of didactics about the natural sciences in the Pedagogy Degree course. (STE-9)

Secondly, there are also STEs who often introduce NOS notions in the context of other content in the school science curriculum:

In the initial training of secondary school [science] teachers, we have a module called ‘Nature and history of science’. (…) [In the context of] physics I usually deal with the heliocentric vs geocentric model (Aristotle, Ptolemy, Copernicus, Galileo, Kepler), Relativity (obtaining evidence from the observation of the eclipse of 1919), the origin of the studies about the Manhattan Project, etc. [In the context of] Chemistry, the chemical elements (from Aristotle to Boyle), early atomistic ideas (Dalton), Lavoisier and the phlogiston theory, the Karlsruhe congress, etc. [These] are introduced through readings and videos, mainly, from which the students have to make explicit reflections on them with the help of question scripts. (STE-1; brackets added)

And thirdly, there are those who introduce NOS content in both an integrated and a non-integrated way with other science content (combined strategy):

Since in most departments [of science in schools] it is decided to start with a topic about scientific methodology, I introduce some NOS content without integrating it. I usually do this by going in some depth into a press release about some recent research (…) even if they are not contents of the [programmed science] curriculum. [But I also treat] [NOS] content in an integrated way. I usually ask questions about NOS included in the same activity about other science content. (STE-2; brackets added)

#### Pedagogical Approaches to Teaching NOS

The STEs were also asked to indicate which pedagogical approach they considered most appropriate when teaching NOS (Table 5). Of the different options that were indicated in the survey, the main one chosen by the STEs (73.2%) was that which would combine the implicit (or indirect) approach and the explicit-reflective one. Only 15.5% of the STEs considered that the most appropriate approach to teaching NOS is the explicit-reflective one. Although in small percentages, there were also STEs who considered that the teaching of NOS should be carried out through an implicit approach (7%) or that both approaches are equally effective for teaching this curricular content (4.2%).

**Table 5:** STEs’ opinions on how to teach NOS

Teaching NOS using an implicit (or indirect) approach	7.0%
Teaching NOS using an explicit and reflective approach	15.5%
Either approach (i.e*.* implicit or explicit and reflective) will be just as effective for teaching NOS	4.2%
Teaching NOS using a combination of the implicit and the explicit and reflective approaches	73.2%

One must start by saying that none of the STEs who answered the open-ended questionnaire alluded to the implicit approach of teaching NOS. All have an explicit NOS teaching perspective with more or less clear allusions to the reflective and that the results of this teaching (and learning) are subject to evaluation. Two of the STEs put it like this:

The approach [that I promote to teach NOS] is fundamentally explicit, associated with different teaching strategies and activities. (…) In any case, these are evaluable questions, like any other, that influence the student's grade. (STE-2; brackets added)

I try to always follow a dialogical, reflective approach, generating discussion in small groups (…) We do experimental work (…) with much discussion on how to interpret [the results] from the initial mental models and how we can change those models based on their testing. (…) With regards to the evaluation (…), I try to ask contextual questions where my students have to reflect on the aspects [of NOS] worked on to argue their position in the face of some situation [related to science] (…). (STE-5; brackets added)

In addition to a reflective approach to understanding aspects of NOS, one of the STEs emphasizes that this can serve as a referent for teachers to promote a particular way of teaching science, i.e. as an analogy of how scientific activity develops:

What summarizes my interventions [about NOS] is to make [the student, future science teacher] reflect that each vision or conceptualization of science implies a specific way of giving science classes and a specific way of learning science. (STE-3: brackets added)

A variety of resources to introduce NOS content are indicated, such as reading news from the press related to science, crime stories, games of inference and enigmas, practical work, and stories from the history of science. This is detailed by some of the STEs:

The starting point [for discussing NOS aspects] can be a text or video document that corresponds to a press release, a popular [scientific] text, or a text about the history of science. (STE-2; brackets added)

If there are interesting pieces of news in the press [about science], these are ideal occasions to address NOS, sometimes related to specific contents of the subject and sometimes not. [I think that] these are topics that may be of interest to the students, even if they are not contents of the official science curriculum. (…) [Also,] some games of inference, the partial reading of a crime story, the analysis of scientific controversies, the viewing of a short film with the resolution of enigmas, etc. (STE-5; brackets added)

(…) [To teach NOS] I use learning scenarios based on the history of science, research scenarios, and debates about current news concerning socio-scientific issues. (STE-4; brackets added)

#### Selection of Content to Teach NOS

**Fig. 5:**
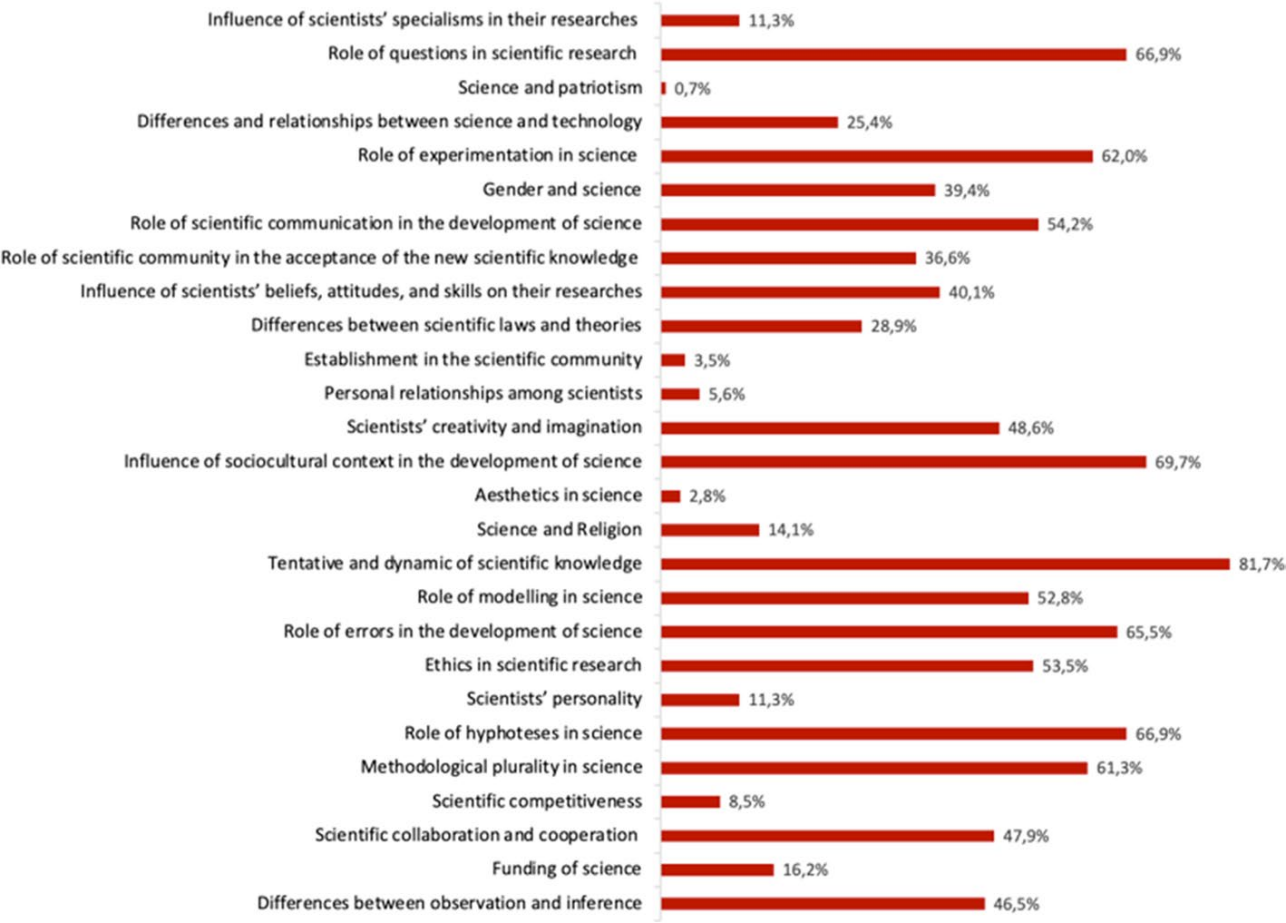
Aspects of NOS selected as priorities by STEs for teaching this content

Finally, there was the intention to determine which NOS content or aspects are considered to be priority by the STEs when planning its teaching. The results of asking them to select 10 of the NOS content topics included in the list they were provided with are shown in Fig. 5. The STEs’ NOS content predilections are very diverse.

With regard to the type of NOS content selected, epistemic aspects predominate (i.e. cognitive or rational aspects of scientific activity). It was found that 47.9% of the STEs selected epistemic aspects exclusively or predominantly, while another 47.9% selected both epistemic and non-epistemic (i.e. sociological, psychological, context) aspects in balanced proportions. Only a small portion of the STEs (4.2%) selected only non-epistemic aspects of NOS.

The “provisional and dynamic nature of science” was the epistemic NOS aspect most frequently chosen by the STEs (81.7%). This was followed at a certain distance by the non-epistemic aspect related to “the influence of the sociocultural context in the development of science” (69.7%) and the epistemic aspects “the role of questions in scientific research” and “the role of hypotheses in scientific research” (both chosen by 66.9% of the educators), as well as the “role of error in the development of science” (65.5%). Instead, the NOS aspects that aroused least interest for the STEs were non-epistemic aspects related to “patriotism in science” (0.7%), the “importance of aesthetics in science” (2.8%), the “establishment existing in the scientific community” (3.5%), and the “personal relationships among scientists” (5.6%), among others. The other NOS aspects, both epistemic and non-epistemic, were chosen by the STEs within a wide range from 11.3 to 62%. Here, aspects such as the “role of experimentation in science” (62%), “methodological plurality in scientific research” (61.3%), the “role of scientific communication in the development of science” (54.2%), or “ethics in scientific research” (53.5%) stand out. Other aspects such as the question of “gender in science” (39.4%) or the “differences between laws and theories” (28.9%) receive more moderate attention.

In addition, most of the STEs who answered the open-ended questionnaire tended to deal with a wide diversity of such content, combining both epistemic and non-epistemic aspects depending on the context. The following are two of their explanations in this regard:

[I usually address] different aspects [of NOS] depending on the case to be treated and the context. I would cite both epistemic and non-epistemic contents. I would highlight some, such as the characterization of scientific knowledge compared to other forms of knowledge, especially [to analyse] pseudo-sciences; the diversity of the methods that scientists use and useful content to characterize the methodology of science. When doing practical work, I introduce epistemic aspects (…). Here the specific contents [of NOS] can be very varied (…). In the approach to socio-scientific aspects, the contents of the nature of science are inherent and (…) vary according to the subject [of science] and the educational level. Other [NOS topics that I usually discuss] are: women in science, science funding, the influence of scientists, groups of scientists and institutions in research, the relationship between science and religion, scientific communication, various aspects of science-technology-society-environment relationships, etc. (STE-2; brackets added)

[I usually discuss with my students the] origin of science, differences between science and non-science, epistemological views (positivism vs new philosophy of science), differences between law, theory, model, hypothesis, facts…; method vs methods of science, non-epistemic variables (subjectivity, biases, interests, personality of scientists, political factors…), science-technology relationships, the role of women in science, internal and external sociology of science. (STE-1; brackets added)

Likewise, one of these STEs once again emphasizes the humanization of science when it comes to selecting the NOS content in their teaching practice in this regard:

The most general criterion that I use is to select those contents [of NOS] that most help to ‘humanize’ scientific activity; those that are most useful to try to help my students see science as another tool to think and act in certain situations and that this attitude helps them live better. (STE-5; brackets added)

Nonetheless, there are two STEs who focus solely, or predominantly, on epistemic aspects of NOS, emanating fundamentally from the philosophy of science:

[I propose] an ‘archaeology’ of the term ‘science’ and a brief discussion of very general aspects of the philosophy of science, such as the theory of explanation and contemporary views of scientific theories. (STE-9; brackets added)

[I usually discuss] the difference between observation and inference, the provisional nature of scientific knowledge, the plurality of the methods of science, the difference between hypothesis-law-theory, the factors that influence the acceptance of new scientific theories, the role of imagination and creativity, and the influence of scientific subjectivity in the future of science (…). (STE-3; brackets added)

### What Correlations Exist Between the STEs’ Preparation, Experience, and Opinions About Teaching and Learning NOS?

The correlation analyses, using Spearman’s *ρ*, show several statistically significant relationships at a significance level of 0.01 between the variables (i.e. preparation, experiences, and opinions of the STEs). The correlation coefficients in this analysis will be classified in the following way (Martínez et al., 2009): strong (*ρ* ≥ 0.76), substantial (0.51 ≤ *ρ* ≤ 0.75), moderate (0.26 ≤ *ρ* ≤ 0.50), and weak (*ρ* ≤ 0.25).

The greatest positive correlation observed, and classified as being substantial (*ρ* = 0.74), relates the “experience in teaching NOS” with the “experience in training science teachers in the didactics of NOS”. In other words, those STEs with more experience in teaching NOS were those who also had high experience in NOS pedagogy training. Likewise, a substantial positive correlation is found, although to a lesser degree (*ρ* = 0.53) than the previous one, between the STEs’ “PCK to teach NOS” and their degree of “experience in teaching NOS”. In other words, the STEs who claim they have a high level of PCK for teaching NOS were the most experienced in teaching this content. There are substantial positive correlations between the “importance that the STEs give NOS in their science teacher training plans” and their experience in teaching NOS (*ρ* = 0.52) and in training science teachers about NOS (*ρ* = 0.54).

With *ρ* values ranging between 0.29 and 0.50, one finds a moderate influence of the variables “level of understanding NOS” and “PCK for the teaching of NOS” about the variables “experience in teaching NOS”, “experience in training science teachers about NOS”, and “importance that they give to NOS in their science teacher training plans”. In other words, possessing an informed understanding of NOS and a high level of PCK when teaching NOS only influences in a moderately decisive way how the STEs prioritize this content in their training plans. In the rest of the cases, the correlations obtained are statistically weak or insignificant.

## Discussion

This exploratory study has attempted to determine what Spanish STEs know, do, and think about teaching NOS. The results offer interesting information that will be discussed in detail in this section.

### What Preparation Do the STEs Claim They Possess Regarding the Teaching of NOS?

Most of the participant STEs estimate their understanding of NOS as being sufficiently well informed. This was to be expected given that they are at the top in terms of their academic status in science education. Nonetheless, this self-estimated understanding of NOS is not inconsiderably weaker than that of their understanding of other school science content. A possible interpretation of this is that they seem to be relatively more confident about their mastery of the latter. This is understandable when one takes into account that, in Spain, NOS has not traditionally been part of the science education plans for the different educational levels (Vazquez-Alonso et al., 2013). Likewise, equally improvable is the attention paid to NOS in science teacher training programs (Acevedo-Díaz & García-Carmona, 2016; Perales et al., 2014; Vázquez et al., 2013), which the STEs themselves are also a product of. The situation may well be similar in other educational contexts. In Turkey, for example, candidates preparing to become STEs arrive with just a limited understanding of NOS (Irez, 2006).

Additionally, a major proportion of the STEs claim to have more than sufficient pedagogical preparation to teach NOS. Such a high self-estimation of Spanish STEs’ capacity to teach NOS is undoubtedly a hopeful piece of information regarding the future of NOS in science education in the country. Nevertheless, some caution is called for since, among other reasons, theirs was an overall evaluation which did not distinguish the various dimensions making up the PCK to teach NOS. It is unknown, therefore, which of these dimensions the STEs were referring to. Neither do the few previous studies on STEs and teaching NOS allow even a minimal discussion of the issue. In the aforecited study of Irez (2006), for example, the focus was on the conceptions of a very small sample of future Turkish STEs about NOS. And the study of Wan et al. (2011) was of just 24 Chinese STEs to analyse what values they consider that teaching NOS contributes to the training of future science teachers.

### What Experience Do the STEs Claim They Possess in Teaching NOS and in Teaching NOS Pedagogy?

#### Experience in Teaching NOS Content and in Training Science Teachers in NOS Pedagogy

Many of the STEs claim to have notable experience in teaching NOS. But the percentages at which this vast experience is expressed are less than those for their (also estimated highly) CK and PCK to teach NOS. Two interpretations are possible. For CK, an explanation for the mismatch could be that some of the STEs have a good understanding of NOS but have not had the chance to teach it (perhaps they do not themselves teach science but instead just science pedagogy). For PCK, the mismatch observed suggests that a broad and adequate understanding of NOS does not always translate into a firm commitment to introduce it into science classes (Akerson & Abd-El-Khalick, 2003; Deniz & Adibelli, 2015; Water-Adams, 2006) and that this is so even among STEs, whom one would assume to be more aware of the importance of NOS in science education.

#### Attention to NOS in Science Teacher Training Plans

According to the recommendations with respect to NOS of the international science education community, it is to be hoped that this topic should occupy a prominent place in any plan for science teacher training. The present results indeed indicate that most STEs state that NOS plays a leading part in their teaching plans. Nonetheless, this supposed strong attention to NOS in the training of Spanish science teachers is not matched by the scarcity of studies published by Spanish STEs on the teaching of NOS (García-Carmona, 2021c). It is also striking that somewhat more than a fifth of the STEs pay scant attention to NOS in their science teacher training plans. The reasons for this may be manifold. Knowing that NOS is thinly served in Spain’s official requirements for school science (Acevedo-Díaz et al., 2017), the STEs perhaps find a compelling reason to be educational conviction (Waters-Adams, 2006), i.e. that their teaching plans should prioritize the content which is most emphasized in the official science curriculum. This may find reinforcement in the fact that, in Spain, NOS content does not form part of any test evaluating pupils’ scientific competence, so that one could well understand that what is not going to be evaluated is not taught (García-Carmona, 2021c). Such a situation is, however, dissonant with the scientific competence assessment test of the international PISA program (Organisation for Economic Co-operation and Development (OECD), 2019) which does evaluate pupils’ understandings of NOS and which Spain has participated in for years.

### What Opinions Do the STEs Have About Teaching and Learning NOS?

#### Educational Stages at Which NOS Should Be Taught

Somewhat more than two-thirds of the STEs are in favour of NOS forming part of school science programs from an early age. This is coherent with positions in this regard defended in the literature (Akerson et al., 2011; Cañal et al., 2016; NGSS Lead States, 2013). While there is some disagreement as to its introduction in pre-primary (3–5 years) or from primary (6–12 years) education onwards, the majority of the STEs consider that NOS should form part of the different educational stages’ science curricula and the rest that it should be taught at specific educational stages (e.g. primary or secondary).

#### Introduction of NOS Content into the School Science Curriculum

For the great majority of the STEs, the best way to introduce notions of NOS into the school science curriculum is as cross-cutting content integrated with the rest of the curriculum. The survey did not ask for educational reasons for the choice of one option or another. Neither did any of the STEs who picked out this option in their responses to the open-ended questionnaire explain what led them to that choice. It remains unknown therefore whether the STEs in this study are aware that integrating NOS with other content can help learners understand scientific knowledge (Driver et al., 1996) or that in this way NOS is discussed in science contexts that are more authentic (Allchin, 2011; Clough, 2006). Perhaps the underlying reason is simply that STEs are aware of how overloaded science education programs tend to be and of how this option can be a good way of favouring the introduction of NOS content (Bell et al., 2012). In any case, this will be a question that new studies will have to go deeper into.

Nonetheless, the other two possibilities for introducing NOS content into the science curriculum (i.e. as stand-alone content or as both integrated and stand-alone content) are also selected by the STEs, although in much smaller proportions. According to the information obtained with the open-ended questionnaire, the main reason for opting for the introduction of NOS as independent content is that many science departments in Spain’s schools establish an initial block on scientific methodology, and this block would include some aspects of NOS. This is, however, a misinterpretation of the Spanish science curriculum since, although it establishes a first generic block called “initiation to scientific activity”, it is suggested that the content of that block be treated in a cross-cutting way with the rest of the content (Education Ministry, 2015, p. 257). Another reason given by some STEs to treat NOS content in a disjointed or independent way, and only at the beginning of the course, is to try to hook their students into the science subject. With this, in some way, it is being assumed that the NOS topics are of second order or supplementary compared with the more classical contents of school science.

#### Pedagogical Approaches to Teaching NOS

It was particularly striking that only a small portion of STEs point to an explicit and reflective approach, i.e. the one recommended by education research on this issue (Lederman, 2007, 2019; McComas & Clough, 2020), as being the best option for teaching NOS. The most widely held view among the STEs is that which would combine the implicit (or indirect) and explicit-reflective approaches to teaching NOS. This result may make it appear that the STEs adopt an eclectic position as the most successful for teaching NOS because it would be assuming the “best” of each approach. Nonetheless, such a position implies recognizing that an implicit approach is also effective in teaching NOS, which enters into clear conflict with the results of empirical research in this regard that has repeatedly proven it to be ineffective as against the explicit-reflective approach (Acevedo-Díaz, 2009; Lederman, 2007). This ignorance of research findings is even more evident in those few STEs who point to an implicit approach being the best way to approach the teaching of NOS or that it is as effective as the explicit-reflective one.

This result therefore once again invites one to accept with extreme caution the self-assessed ample PCK to teach NOS that most of the STEs declare on the third item of the survey. It also allows one to deduce an undesirable consequence: that the majority of future Spanish science teachers are probably receiving training that advocates implicit teaching about NOS. And this, in accordance with what educational research on this subject suggests (Acevedo-Díaz, 2009; Khishfe & Abd-El-Khalick, 2002; McComas & Clough, 2020), will do little to help improve the teaching, and therefore understanding, of NOS in Spanish science classrooms.

The open-ended questionnaire confirmed that the STEs who support the explicit-reflective approach understand well what it means to teach NOS in this way. They explain that, within the framework of an approach like this, they propose activities for their students involving specific questions for thinking about and discussing aspects of NOS, that they propose ad hoc evaluation activities, and that they use different resources such as readings from the history of science, scientific news in the press, school-level inquiry activities, enigma games, etc. for their students to reflect on aspects of NOS, etc. These are resources and activities that are widely recommended in the recent literature on teaching NOS. Likewise, one of the STEs aligned with the explicit-reflective approach argues that a science teacher’s own form of conceptualizing NOS marks a particular way of teaching science in general. This is in tune with the idea that an informed understanding of NOS can help promote science learning situations that are more in accord with authentic scientific practice (Abd-El-Khalick, 2013; García-Carmona & Acevedo-Díaz, 2018).

None of the STEs responding to the open-ended questionnaire, however, align themselves with the implicit approach. It is therefore impossible to know what the STEs in favour of this approach really have in mind when they think about NOS teaching. Be that as it may, it is hard to understand how NOS teaching can be planned without considering the need to design specific activities so that the students can put their minds to reflecting on questions about this complex and multifaceted content.

#### Selection of Content to Teach NOS

As for the content of NOS selected by the STEs, overall there predominate that of an epistemic nature (i.e. rational or cognitive aspects of scientific work). Likewise, there are two fairly balanced majority trends among the STEs. On the one hand, there are STEs who choose only or predominantly epistemic aspects of NOS. Possibly, they are strongly influenced by the dominant primary approaches to NOS in science education (e.g. the seven tenets of NOS, Lederman, 2007). And, on the other, there are STEs who select epistemic and non-epistemic aspects in similar proportions, reflecting an alignment with the most recent positions that promulgate a broader, holistic vision of NOS (Allchin, 2011; Dagher & Erduran, 2016; García-Carmona & Acevedo-Díaz, 2018). Among those who align themselves with this latter perspective, there are STEs who use it as a criterion for selecting NOS content that serves to “humanize science”.

It should be said that it is educationally legitimate to opt for either of the two visions regarding the selection of NOS content, since what is really essential is that a conscious and effective teaching of NOS is being projected in the terms that have been discussed above. Nonetheless, the aforecited most recent approaches to NOS teaching argue that attention to non-epistemic aspects can offer a broader and richer view of how science really works. As well as presenting a truer image of science, this can favour the inclusion of NOS topics in science classes because teachers will have a wide range of possibilities to choose from. In line with this vision, therefore, it would be desirable for all STEs to consider promoting both epistemic and non-epistemic aspects when training future science teachers about NOS and its pedagogy.

### What Correlations Exist Between the STEs’ Preparation, Experience, and Opinions About Teaching and Learning NOS?

The correlation analyses provide some notable results concerning the influences interconnecting the STEs’ opinions and performances regarding the teaching of NOS. For instance, the STEs most experienced in teaching NOS are those who also declare the highest level of PCK on the topic and extensive experience in training future teachers in pedagogy about NOS. This highlights how important it is that an STE has previously been a teacher of what they are now trying to train other teachers to do — in this case, teaching NOS. This is doubtless the desirable STE profile with which to favour reducing the gap between theory and current practice in teaching NOS (Ferreira & Morais, 2013; Höttecke & Silva, 2011). Nonetheless, this declared broad PCK to teach NOS should be accepted with some reservation, as well as bearing in mind that experience and expertise do not always go hand in hand.

That those STEs with experience in teaching NOS are interested in integrating it into their teacher training plans is surely due to an educational conviction that understanding NOS is an essential part of adequate scientific literacy for today’s citizens (NGSS Lead States, 2013; Organisation for Economic Co-operation and Development (OECD), 2019). In this sense, Wan et al. (2011) argue that a good way to convince science teachers to teach NOS is, in teacher training plans, to promote an understanding of the values that teaching NOS can contribute to people’s integral education.^2^ Therefore, in plans for science teacher education on NOS, the results and recommendations of educational research in this regard should be prioritized instead of strictly adhering to what is suggested by the official prescriptions for school science, since the latter tend to lag significantly behind the former (Acevedo-Díaz et al., 2017; Olson, 2018).

One finds that the fact that an STE has an informed understanding of NOS and a high level of PCK to teach it is not a decisive influence for them to give it a leading role in their science teacher training plans. Also involved in their decisions about teaching NOS are complex and diverse factors that go beyond the mere fact of a more or less mastery of the school-level content (Akerson & Abd-El-Khalick, 2003; Deniz & Adibelli, 2015; Waters-Adams, 2006), and this reiterates the importance of STEs’ reflecting on what their priorities should be in fostering comprehensive scientific literacy through the future science teachers they are training.

## Limitations and Perspectives

This research adds to the few studies (e.g. Irez, 2006; Wan et al., 2011) that have addressed the preparation, experiences, and visions of STEs in relation to teaching NOS. Those studies were conducted with small samples of STEs from two particular contexts (Turkey and China). The present study was carried out with a representative sample of Spanish STEs. While it is true that the participating STEs also belong to a specific educational context (Spain in this case), the sample’s representativeness possibly makes the extrapolation of some of the results and conclusions to other geographical contexts of science teacher training with similar characteristics more reliable.

The analyses carried out provide revealing information on the state in Spain of science teacher training in teaching NOS. Nonetheless, as for any study of these characteristics, it is convenient to comment on its limitations and perspectives for future studies. The limitations derive mainly from the research instruments used. With a clear purpose of getting the survey completed by as many STEs as possible, it was designed with relatively few items. While this undoubtedly had the advantage that a large sample of STEs finally participated, it had the disadvantage of it being impossible to delve further into certain aspects of NOS and its pedagogy. In order to complement the information and look in greater depth into certain aspects, an open-ended questionnaire was prepared, although just a small group of STEs were asked to complete it. Even so, the responses provided invaluable information to help understand certain trends or positions observed in the survey, although it was not possible to go any deeper into others. A good way to have compensated for this limitation would have been to conduct semi-structured interviews in which one could have questioned and re-questioned the STEs so as to get more comprehensive and detailed responses. This option had to be ruled out, however, due to the difficult circumstances in which all Spanish teachers, including STEs, found themselves at the time of the research, marked by the global COVID-19 pandemic.

Therefore, in view of the new questions and doubts that arose after the discussion of the results, it would be interesting for future research to address the following aspects among others:

i Although most STEs declared that they have a high level of PCK to teach NOS, a good part of them assumes — contrary to the empirical evidence — that an implicit approach is at least as effective as the explicit approach. Therefore, in a future study, it would be interesting to determine what the STEs understand by an implicit approach that encourages learning about NOS and to analyse the effectiveness of some teacher training course in which such an approach to learning NOS is promoted.
ii Another important question will be to determine the STEs’ opinions and reasons about what would be the best option to integrate NOS content into the science curriculum — whether cross-cutting and integrated with the rest of the science content or as specific content independent of the rest.
iii As well as the above, it would be necessary to go deeper into the characteristics of the STEs’ PCK to teach NOS, i.e. to analyse in detail its different basic dimensions (e.g. the purposes for teaching NOS, NOS content, students’ learning of NOS, methods and approaches in teaching NOS, evaluation of NOS, self-efficacy to teach NOS). This would need to be done with small samples of STEs, through case studies, for example.
iv The participating STEs selected a diversity of aspects of NOS, covering both epistemic and non-epistemic. Nonetheless, it would be interesting to delve further into this question, for example, analysing which aspects of NOS the STEs consider to be more appropriate to each educational level or the school science content involved if an integrated approach to teaching NOS is chosen. In addition, it would be relevant to investigate whether the STEs incorporate epistemic and non-epistemic aspects of NOS when they train prospective science teachers in NOS teaching and whether they do it implicitly or explicitly.
v It would also be opportune to know what STEs’ opinions are about the reasons behind NOS’s continuing lack of presence in science classes in Spain and what proposals they would make to improve this situation with a view to fostering a more comprehensive scientific literacy of the country’s citizens. In this same framework, it would be equally interesting to understand why NOS receives so little attention in Spanish STEs’ education research when a significant fraction of them state that they give NOS high relevance in their science teacher training plans.

## Footnotes

1 According to the review by Acevedo-Díaz (2009), the positions which defend the implicit approach to learning NOS mainly emerged during the 1970s and 1980s. Nonetheless, in the 1990s, there were still some supporters of this implicit approach, despite the fact that at the time there had already been numerous empirical studies that confirmed its ineffectiveness (e.g. Moss et al., 1998; Riley, 1979; Meichtry, 1992; cited in Acevedo-Díaz, 2009, p. 359).

2 Wan et al. (2011) distinguish between the value of NOS within science teaching and its value beyond science teaching.
